# Supplementary material for: Effects of Brown Seaweed (Ascophyllum nodosum) Supplementation on Enteric Methane Emissions, Metabolic Status and Milk Composition in Peak-Lactating Holstein Cows
Source: Animals (Basel). 2024 May 21;14(11):1520. doi: 10.3390/ani14111520 (PMC11171174; doi:10.3390/ani14111520)
Supplement: Supplementary file 1 [file animals-14-01520-s001.zip › animals-2975560-supplementary.pdf]

Table S1. Mean±SE of the values for general characteristics and production of selected cows.

Parameter	Group of cows[1]		
	CON	BS_{50}	BS_{100}
Parity	2.2±0.17	2.2±0.17	2.2±0.17
Days in milk	43.2±5.9	45.8±8.5	45.2±4.6
Milk yield (kg)	42.0±2.8	45.5±3.6	42.5±3.3
Body weight (kg)	586.7±25.9	584.7±18.1	572.2±24.6
Body condition score	2.63±0.09	2.79±0.12	2.67±0.05

[1]CON – control group of cows; BS_{50} – a group of cows supplemented with 50 mL of brown seaweed (10% *A. nodosum*, 10%); BS_{100} – a group of cows supplemented with 100 mL of brown seaweed (10% *A. nodosum*).
